# Desalination of Produced Water by Membrane Distillation: Effect of the Feed Components and of a Pre-treatment by Fenton Oxidation

**DOI:** 10.1038/s41598-019-51167-z

**Published:** 2019-10-18

**Authors:** Francesco Ricceri, Mattia Giagnorio, Giulio Farinelli, Giulia Blandini, Marco Minella, Davide Vione, Alberto Tiraferri

**Affiliations:** 1 Department of Environment, Land and Infrastructure Engineering, Politecnico di Torino, Corso Duca degli Abruzzi, 24, 10129 Torino, Italy; 2 CleanWaterCenter@PoliTo, Politecnico di Torino, Corso Duca degli Abruzzi, 24, 10129 Torino, Italy; 3 Department of Chemistry, Università degli Studi di Torino, Via Pietro Giuria 7, 10125 Torino, Italy

**Keywords:** Chemical engineering, Chemical engineering, Pollution remediation

## Abstract

The treatment of produced waters (by-products of oil and gas extraction) with the innovative process of membrane distillation is challenging, because these highly saline streams contain high concentrations of organic compounds and hydrocarbons that cause membrane wetting and impairment of performance. To design the most compact treatment scheme and with the aim of obtaining an easier management of produced water for reuse purposes, Fenton oxidation is here investigated as a feed pre-treatment that may produce an effluent easily handled by membrane distillation. In high-recovery membrane distillation tests, we systematically investigate the detrimental effects of individual contaminants in a synthetic produced water mimicking the composition of a real sample. The recovery rate depends strongly on the initial salinity, which eventually causes scaling and pore blocking. Surfactants are found to be mainly responsible for membrane wetting, but volatile and hydrophobic organics also spoil the quality of the product water. A Fenton oxidation pre-treatment is thus performed to degrade the target organics, with the aim of enhancing the effectiveness of the following membrane distillation and to improve the quality of the final product. The combined oxidation-membrane distillation scheme has both advantages and limitations, which need to be carefully evaluated and further investigated.

## Introduction

In oil & gas extraction activities, large amounts of water are injected into the reservoir to counteract the subsoil pressure and achieve high recovery levels. As this fluid resurfaces, it is enriched in valuable hydrocarbons and is referred to as “produced water”^1^. The global production of produced water is estimated at 250 million barrels per day, with an increasing trend^2,3^. This stream contains various organic and inorganic constituents at variable concentrations^4^, which calls for a complex design of cost-effective, flexible, and low-footprint treatment plants to separate the oil and to obtain a water effluent with sufficient quality^2,5^. This problem is further exacerbated by the growing need to recycle the product for re-injection or reuse for beneficial purposes, e.g., irrigation, and in offshore applications, where space is limited and compactness is required^6^. The discharge of the treated water is of environmental concern for both soil and water, thus it is strictly regulated^7,8^.

Many efforts are currently focused on evaluating processes and schemes to improve the management of produced water, which would bring about tremendous environmental and economic benefits. For example, Tsang and Martin^9^ reported a system including dissolved gas flotation, walnut shell filtration, and softening followed by membrane bioreactor and reverse osmosis. Doran *et al*.^10^ proposed another treatment train including precipitative softening to remove silica, boron, and part of the hardness, followed by organics and ammonia removal using a biological trickling filter. These steps were followed by filtration and ion-exchange softening, before the final reverse osmosis desalination. Other authors investigated the efficiency of coagulation, adsorption, ultrafiltration, and reverse osmosis for the treatment of shale gas produced water from the Sichuan basin^11,12^.

When compactness, flexibility, and modularity are important, such as in the case of produced water management, membrane-based processes are highly promising^13^. The limitations of these technologies are mostly related to fouling, which discourages their application without extensive primary treatments^13^. The latter usually include sedimentation, coagulation, flocculation, and flotation^14^. Considering the final desalination step, reverse osmosis systems cannot treat feed streams with concentrations of solutes above 70 g/L^15,16^. Such levels are easily reached in most produced waters, especially from wells late in their lifetime of production. Membrane distillation (MD) is an emerging thermally-driven membrane process with great potential for the treatment of produced water, because it can achieve high water recovery from feeds of virtually any salinity^17^. This process may be especially competitive when low exergy heat is available as energy source: this is the case of many oil & gas extraction fields, where stream temperatures can exceed 100 °C due to geothermal heat^18–20^. Despite of its potentially promising characteristics, the MD technology still needs to be optimized for produced water desalination, and in particular to overcome the detrimental effects of wetting induced by the complex feed streams^21,22^.

Membrane wetting can be described as the progressive loss of hydrophobicity caused by compounds able to decrease the surface tension^23^, such as organics (especially those with amphiphilic nature) and salt crystals. While these phenomena have been observed in many studies, the literature comprises contradictory reports concerning the extent and the mechanism of wetting, as well as the onset and consequences of scaling^24,25^. Few MD experiments have been performed thus far with the complex mixture that characterizes produced water streams, and few as well had the aim of achieving high water recovery rates. Moreover, no compact and cost-effective pre-treatment has been identified to tackle membrane wetting in MD when managing produced waters. Interesting reports are available that discuss the use of forward osmosis (FO) as a pre-treatment for MD, although these processes are not yet at a commercial readiness level. Therefore, they represent a promising but still impractical option in the near future^26,27^.

In this study, the performance of MD is discussed for the treatment of synthetic feeds mimicking produced water effluents from primary treatment and containing a complex mixture of organic compounds and dissolved solutes. The first goal is to understand the behavior of the MD process in terms of productivity and product quality with synthetic feed streams containing different constituents; the second objective is to propose a tailored pre-treatment that should be simple to be implemented and effective to enhance the MD performance. Fenton oxidation is here evaluated as a possible innovative solution to remove the organic components causing detrimental effects in MD^28^. Effluents from a Fenton oxidation step are thus fed to the MD system and the potential of this combined scheme is scrutinized by highlighting both its advantages and present limitations.

## Results and Discussion

### Effect of different organic components

**Figure 1:**
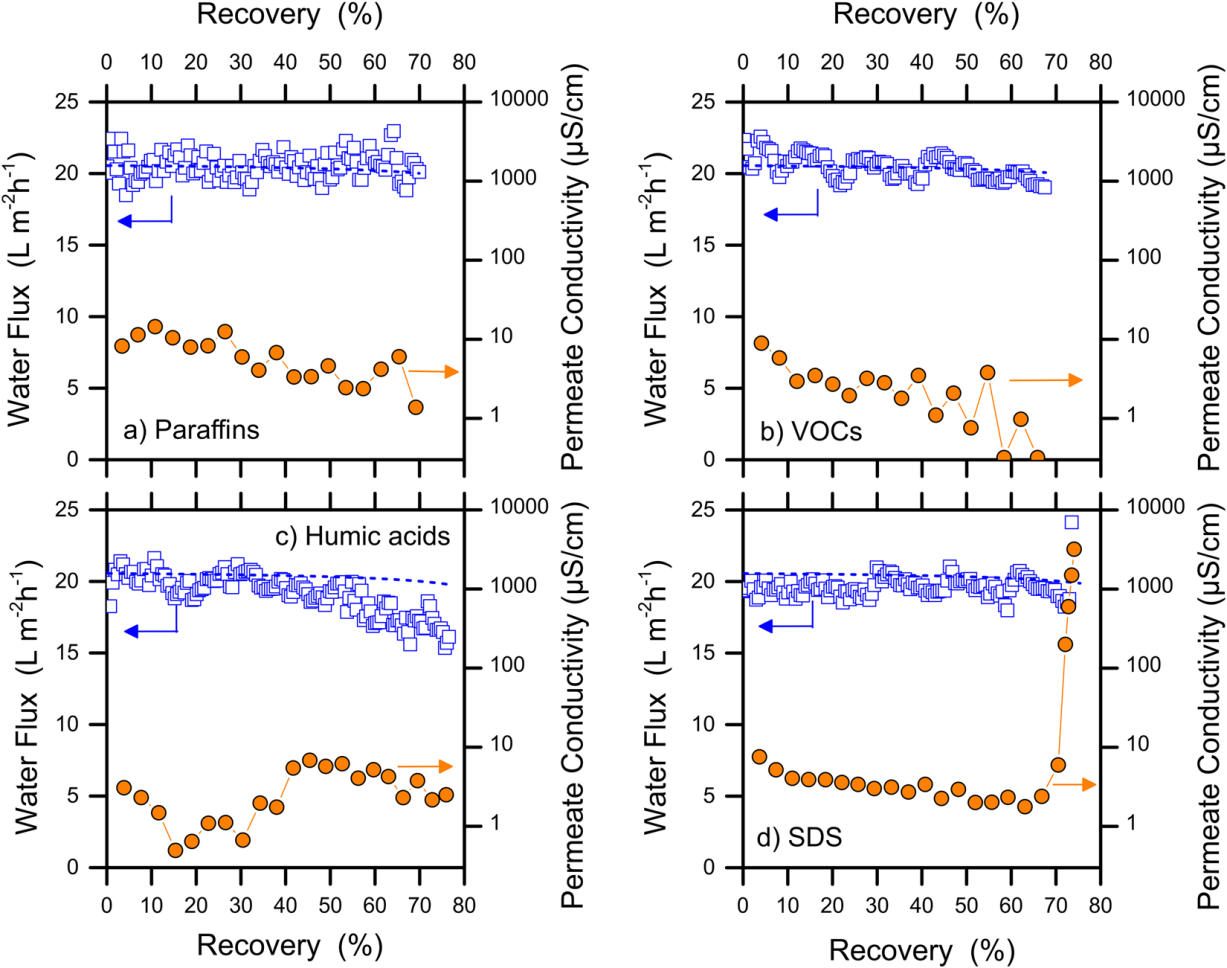
Results of MD filtration tests at high water recovery with feed water composed by individual organic compounds, namely, (**a**) paraffins (mixture of C_n_ saturated hydrocarbons, with n > 25), (**b**) VOCs (volatile organic compounds, i.e., a mixture of p-xylene, benzene, toluene, and MTBE), (**c**) humic acids, and (**d**) SDS (sodium dodecyl sulfate). Blue open squares represent the experimental water flux (left Y-axis), while orange solid circles refer to the permeate conductivity (right Y-axis). The model for water flux is depicted by a blue dash line. For all the feed solutions, the initial concentration of TDS was 15 g/L and the nominal TOC was 800 mgC/L, although a two-phase oil-water system was produced when non-miscible compounds were used (e.g., paraffins). All the tests were conducted at a feed temperature of 50 °C and a distillate temperature of 25 °C, using PTFE membranes.

The study of the wetting behavior of the MD membrane when challenged by different organic compounds may help with the design of the best pre-treatment scheme to maximize the efficiency of this innovative desalination process. Figure 1 presents the results of filtration tests performed to understand the behavior of the system with various individual organic components. All the membranes showed fluxes that were consistent with theoretical expectations in the beginning of each test. Please note that the modeled flux represents the change in productivity that is expected only because of the loss in driving force due to increased salt concentration in the feed solution at increased recovery. The slight oscillation of the water flux profiles in the graphs is mostly attributed to the heating cycles of the feed solution by the intermittent heat exchanger. The presence of organic compounds caused diverse detrimental effects on the filtration performance in the course of the tests.

In detail, no significant flux decline was observed in the presence of paraffins or volatile organic compounds (VOCs); see Fig. 1a,b. The permeate showed a continuously decreasing conductivity, implying an increasing improvement of the purity of the water in the distillate tank. This result also suggests no obvious consequences on membrane wetting, i.e., no salt leakage; however, both paraffins and VOCs were found at high concentration in the distillate tank. Evidence of this phenomenon is presented in Fig. S2 of the SI, showing the relatively high levels of the permeate TOC. Specifically, VOCs were observed to instantly contaminate the distillate product, consistent with their volatile nature. In contrast, paraffins may diffuse to the distillate tank due to their high affinity with the hydrophobic membrane material, in agreement with the low contact angle measured for this substance (51 degrees). A slight decline of the water flux was observed in the presence of humic acids, which can be rationalized with their deposition onto the membrane surface that results in fouling^29^. Finally, SDS had important wetting effects that produced a sudden increase of conductivity (i.e., salt concentration) in the permeate stream above approximately 70% recovery (Fig. 1d). Surfactants are amphiphilic compounds that can lower the feed solution surface tension, thereby allowing the passage of liquid water with its dissolved salts within the membrane pores^30,31^. In such a case, the wetting kinetics is controlled by the rate at which the pore surface is saturated by the adsorbed surfactant molecules^24^. Once the membrane is wetted, the MD process is no longer governed by the trans-membrane vapor tension difference but is instead controlled by the hydraulic pressures.

**Figure 2:**
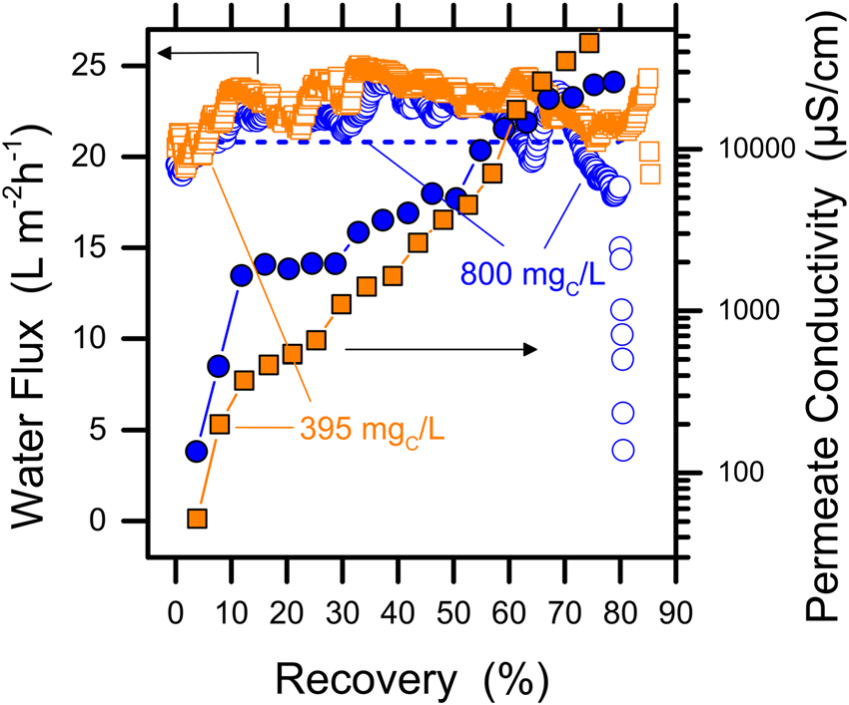
Results of MD filtration tests at high water recovery with feed water composed by paraffins, SDS, and 15 g/L NaCl. Water flux data are depicted by open symbols and refer to the left Y-axis. Permeate conductivities are represented by solid symbols and refer to the right Y-axis. Orange squares and blue circles depict the data obtained with a theoretical TOC concentration of SDS of 395 mgC/L and 800 mgC/L, respectively (equivalent to SDS concentrations of 2.7 mM and 5.5 mM, respectively). The model for water flux is depicted by a blue dash line. Paraffins were added into the feed water at an amount of 1000 mgC/L, but this component is only sparingly soluble in water. All the tests were conducted at a feed temperature of 50 °C and a distillate temperature of 25 °C, using PTFE membranes.

A common question among researchers is what happens when both oil (here represented by paraffins) and surfactants (here modeled with SDS) occur together in the feed water, as the two components interact. The adopted SDS concentration was lower than its critical micelle concentration (CMC, ~8.2 mM = 2.4 g/L)^32^; therefore, the organic compounds were not sequestered in the hydrophobic core of SDS micelles, but strongly interacted with the lipophilic tails of the surfactant. The results presented in Fig. 2 suggest that wetting by SDS occurred immediately even when paraffins were dispersed in the feed water. This observation does not corroborate the hypothesis that the co-occurrence of oils and surfactants would result in reduced or delayed wetting compared to the presence of the two components free in solution. Our results are in agreement with the findings of Han *et al*.^33^, who proposed that the presence of sodium chloride in the feed solution decreases the surfactants-oil interactions, thus allowing that the two compounds greatly impair the process integrity with no delay or obvious lessening of the wetting process.

The results presented so far suggest that, at medium salt concentrations, wetting and fouling by organic compounds may not affect directly the water productivity of the MD process. This finding is opposite to typical observations with pressure-driven membrane processes applied to similar aqueous streams^34,35^. However, because the quality of the product (i.e., the permeate) may be significantly spoiled during the tests, suitable pre-treatments for the desalination step by MD should remove volatiles, oil, and grease. Due to their important wetting effects, any trace of surfactants should be eliminated as well^29^. Interestingly, the presence of humic acids was associated with significantly lower detrimental effects, compared to the other organic compounds.

### Effect of TDS concentration and importance of antiscalants

**Figure 3:**
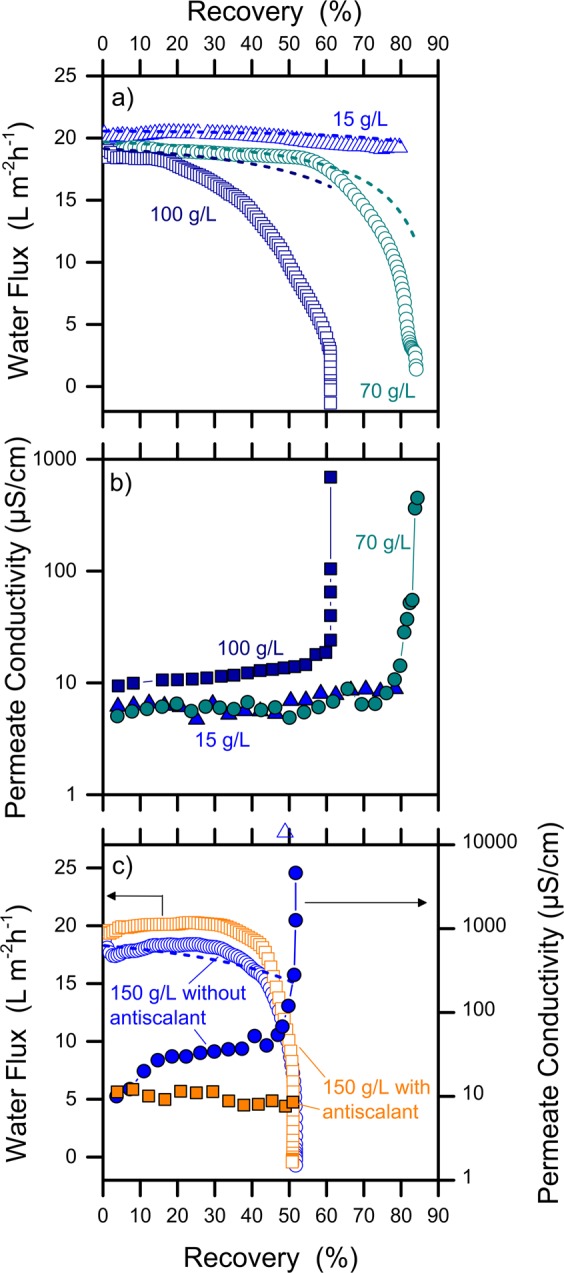
Results of MD filtration tests at high water recovery with feed water containing different concentrations of NaCl, namely, 15, 70, 100, or 150 g/L. (**a**) Experimental (open data points) and modeled (dash lines) water flux; (**b**) permeate conductivity; (**c**) water flux and permeate conductivity with a feed of 150 g/L NaCl with and without antiscalants. All the tests were conducted at a feed temperature of 50 °C and a distillate temperature of 25 °C, using PTFE membranes. A 4-L feed volume was used for these experiments.

The level of TDS in solution influences the formation of salt crystals, i.e., scaling, during the MD process. Due to the intrinsic variability of TDS in produced waters, experiments were performed with different initial NaCl concentrations in the feed solution, namely, 15, 70, 100, and 150 g/L. No substantial flux decline was observed with a feed solution containing 15 g/L NaCl (Fig. 3). This finding indicates no significant scaling during the timeframe of the test and for a recovery rate up to 80%. As the initial NaCl concentration increased, inorganic fouling (i.e., scaling)^36^ was observed with a consequent quick drop of membrane productivity (Fig. 3a) and a concurrent rise of permeate contamination by salt. The onset of this phenomenon can be identified in Fig. 3b as a sharp growth in permeate conductivity, which occurred at lower recovery rates for higher TDS concentrations in the feed. In such a case, the experimental data departed from the flux model sooner in the course of the test. According to a simple calculation based on the recovery rates and reported in Fig. S5 of the SI, the bulk feed concentration of NaCl at the end of the tests was on average 285 g/L, which is slightly below the NaCl water solubility. However, the salt concentration at the membrane interface is higher than that in the bulk due to concentration polarization^37^, increasing the likelihood of crystallization. The fact that the convective water flux goes to zero while the salt flux increases is ascribed to scaling-induced wetting, because NaCl crystals reduce membrane hydrophobicity and let liquid water leak through the pores^25,38–40^.

The results reported so far imply that the recoverable product water from a hypersaline feed is a strong function of the initial salinity; for example, a maximum recovery of roughly 50% was observed for an initial feed of 150 g/L NaCl under the operating conditions of this study. Experiments were also performed by adding polyacrylates in solution, chosen because common anti-scaling agents and especially effective in the prevention of gypsum scaling. While no remarkable improvement of membrane productivity was achieved compared to the case without antiscalants, the quality of the product was greatly improved. Indeed, the constant value of permeate conductivity throughout the test with antiscalants contrasts with the sharp conductivity increase observed above 40–50% recovery when antiscalants were not used; see Fig. 3c. Possibly, the presence of antiscalants delayed or reduced the deposition of crystals within the membrane pores, although they did not prevent pore blockage at the membrane interface. It is important to notice that NaCl is characterized by high water solubility. Normally, scaling in MD is attributed to other non-alkaline cations, such as Ca or Mg, which are more likely to precipitate as carbonates, or as CaSO_4_, MgCl_2_, MgSO_4_, and for which antiscalants are usually more active than with NaCl^36,40^. On the other hand, when anthropic streams are treated by MD, high NaCl concentrations represent a major concern at high recovery rates^41^. Overall, these results corroborate the initial assumption that MD is a promising technology to extract high-quality water from hypersaline feeds^42,43^. With the goal of obtaining water for reuse purposes, addition of antiscalants is strongly recommended for initial TDS concentrations higher than 40–45 g/L (Fig. S5). However, antiscalants should be used at low doses to avoid potential membrane wetting due to their organic nature^21^. When treating real produced water, some of the organic contaminants that can already be present in the feed solution may act like antiscalants inhibiting the formation of crystal nuclei; these phenomena have been poorly understood. Further research should focus on the improvement of MD performance under these challenging conditions, also to maximize the recovery rates.

### Coupling of Fenton oxidation and membrane distillation for the reuse of produced water

**Figure 4:**
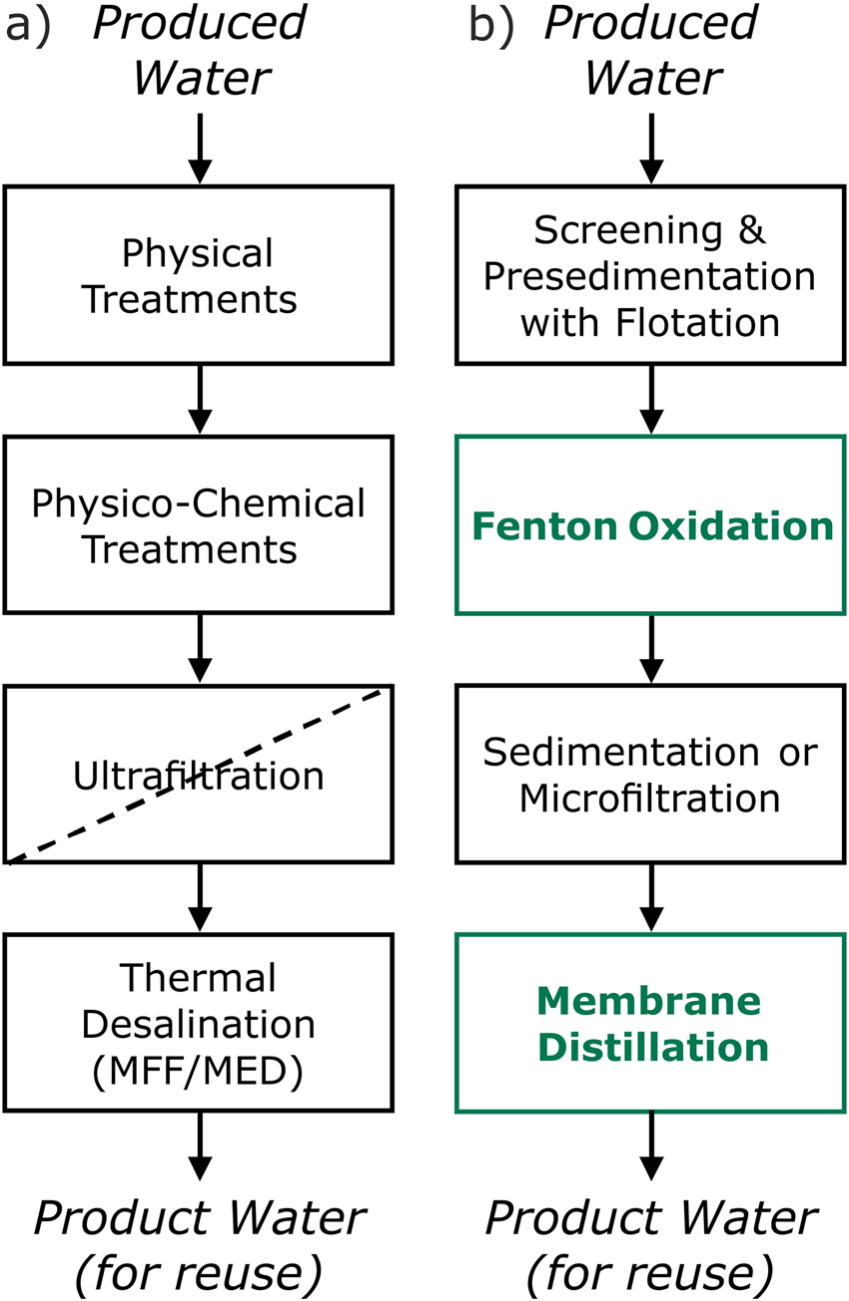
Potential treatment schemes for the beneficial reuse of flowback and produced water, including the compact scheme achieved by coupling Fenton oxidation and membrane distillation (MD). In (**a**), physical treatments usually include the screening of large contaminants followed by pre-sedimentation with flotation and possible additional oil separation and stripping; physico-chemical treatments usually include coagulation-flocculation and settling, as well as possible further treatment by depth filtration/adsorption. In (**b**), these steps and the ultrafiltration step may be substituted with Fenton oxidation, followed by sedimentation with flotation or microfiltration.

Currently, conventional treatment schemes for produced water management consist of a wide array of separation processes, due to the complexity of the influent stream. Typical schemes include primary treatments made up of primary settling with flotation, oil removal, and stripping, followed by physico-chemical treatments, such as coagulation, flocculation and settling. These are usually combined with activated carbon or a depth filtration step, followed by ultrafiltration systems for the abatement of turbidity and organics before the final desalination. The latter is mostly performed through thermal processes or electrodialysis (Fig. 4a)^4,14,18,44^. In some cases, and mainly for onshore applications, biological treatment is also included and is coupled with a secondary sedimentation system^45,46^. These current schemes do not fulfill the requirement of compactness, especially required in offshore sites. The results discussed so far and those available in the literature suggest that MD may become a feasible technology for water extraction from produced wastewater, but it requires a series of preliminary steps to remove the various contaminants that may affect the process performance. This study suggests that surfactants, oil & grease, as well as volatile compounds are mostly responsible for MD performance impairment that lowers both the quality of product water and the maximum recovery. Advanced oxidation processes (AOPs) are a family of innovative techniques aimed at efficiently degrading organic contaminants, and particularly the biorecalcitrant ones, from aqueous streams with diverse characteristics^47–50^. The Fenton reaction is a promising example of AOP that can be applied with reasonable flexibility to a wide range of organic contaminants^51^. One of the key advantages of this method is that it degrades the contaminants rather than merely separating them from the main stream, and it may potentially substitute various physico-chemical treatments. The Fenton reaction involves the addition of iron and H_2_O_2_ in an acidic medium (pH ~3), followed by separation of iron hydroxide flocs in a settling or microfiltration unit after pH neutralization. In this way, it combines chemical transformation with the possibility to perform a further polishing/separation step of separation through flocculation, settling, and flotation, before MD desalination. In Fig. 4, we compare the conceptual schemes of a traditional treatment train (4a), and of a system that combines the Fenton reaction with the MD technique (4b), to manage the synthetic water described in Table 2.

**Table 1:** TOC, conductivity, and (where applicable) recovery rates in the main feed waters and in the permeate streams for each treatment step.

Parameter	Synthetic produced water			Synthetic produced water without SDS		
	As is	Oxidized	AVG of MD permeate in the case of oxidized feed	As is (feed)	Oxidized	AVG of MD permeate in the case of oxidized feed
Recovery		>95%	70%^c^		>95%	65%^c^
TOC (mg/L)	444^a^	442^a^	88.8^b^	495^a^	354^a^	20.1^b^
Conductivity (µS/cm)	124,200^a^	95,300^a^	475^b^	106,700^a^	109,600^a^	9.9^b^

The permeate quality is computed as the average of the entire high-recovery tests.

^a^Measured.

^b^Computed from mass balance.

^c^Maximum achieved recovery during the test.

**Table 2:** Composition of the synthetic wastewater used in this study, compared with the reference real effluents.

		Synthetic produced water		Real produced water
Parameter	Component	Concentration (ppm)	Equivalent TOC (ppm)	Concentration (ppm)
TOC	Paraffins^58^	200	not dissolved	–38856
	Surfactant (SDS)^4,58^	790 [2.7 mM]	395	
	Humic acids^58^	500	195	
	Naphthalene^4^	1	1.2	
	Cyclohexane^56^	2	1.8	
	Phenol^56^	2.5	1.9	
	P-xylene^56^	1	1	
	Benzene^56^	12	11.3	
	Toluene^56^	4	3.4	
	MTBE^56^	260	178	
	**TOT**	**1,772.5**	**788**	**Maximum 500** ^4^
TDS	Sodium chloride	98,870		Typically, 38,500–238,000^56^
	Sodium sulfate	5,270		
	Sodium bicarbonate	853		
	**TOT**	**105,000**		**Avg. 100,000** ^57^

The synthetic water composition includes representative pollutants of a flowback and produced water effluent, to mimic typical for TOC and TDS values of the reference matrices.

**Table 3:** Characteristics of the porous PTFE membrane.

Data source	Parameter	Symbol	Units	Value
Provided by the manufacturer	Thickness	δ	µm	77
	Mean pore size	d	µm	0.17
	Porosity	ɛ		0.83
	Thermal conductivity	*k*_*m*_	J m^−1^K^−1^	0.038
From experiments	Water contact angle	CA_w_	Deg	127
	Petroleum jelly (paraffins) contact angle	CA_v_	Deg	51
	Membrane permeability coefficient	*b*	L m^−2^h^−1^bar^−1^	226

### Advantages and drawbacks of coupling Fenton oxidation and membrane distillation

**Figure 5:**
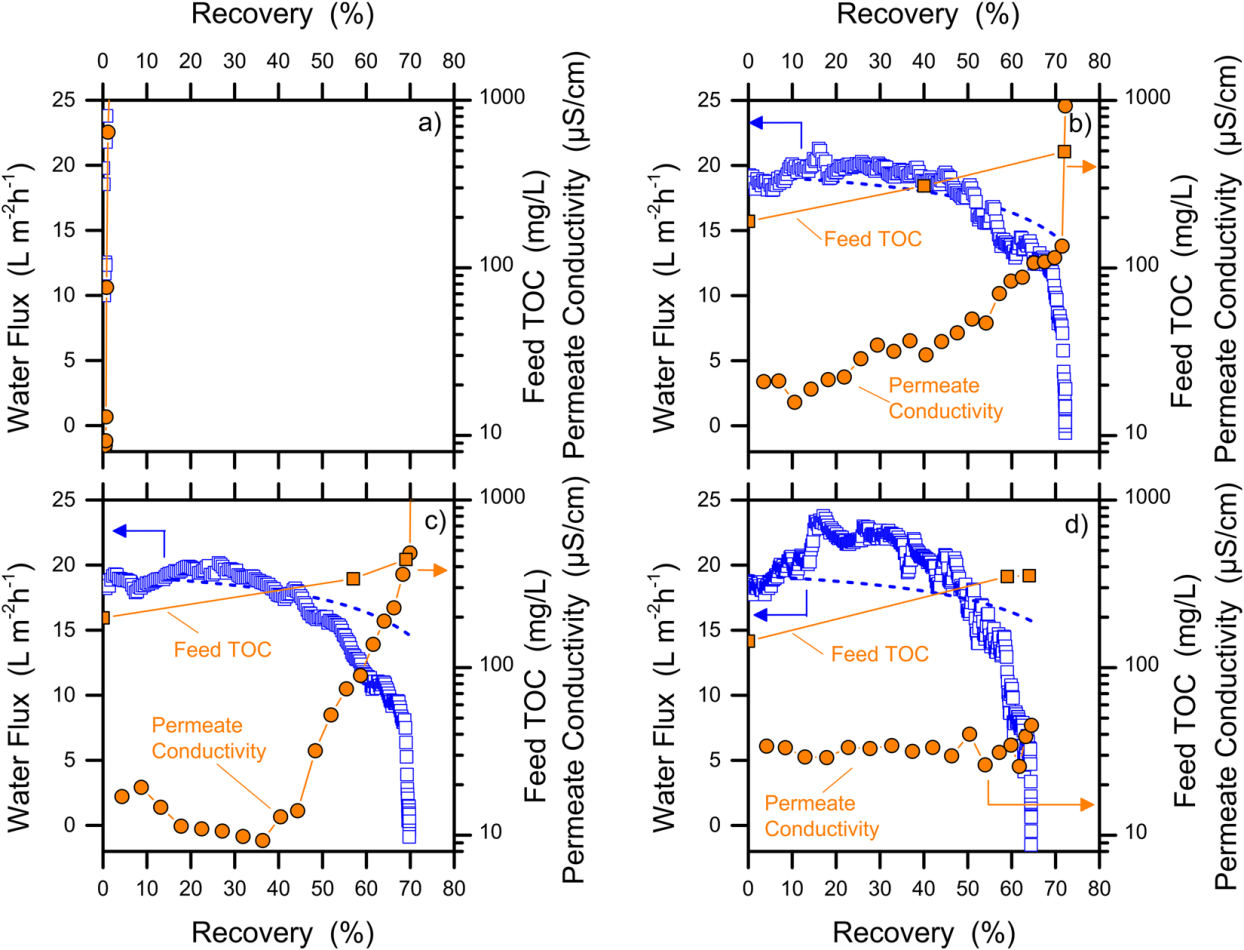
Results of MD filtration tests at high recovery with synthetic feed water mixtures. The feed waters in the different graphs were as follows: (**a**) synthetic water, (**b**) synthetic water without SDS, (**c**) synthetic water after Fenton oxidation, (**d**) synthetic water without SDS after Fenton oxidation. Blue open squares represent the experimental water flux (left Y-axis), while orange solid circles and squares refer to the permeate conductivity and feed TOC concentration, respectively (Y-right axis; note the double measure unit). The model for water flux is depicted by a blue dash line. The composition of synthetic waters is summarized in Table 2. All the tests were conducted at a feed temperature of 50 °C and a distillate temperature of 25 °C, using PTFE membranes.

When the synthetic water (see Table 2 for its composition) was used as feed solution directly for the MD treatment, the test was utterly unfeasible and immediate wetting was observed in several replicate experiments. This result is apparent in Fig. 5a, which shows that both water flux and permeate conductivity increased rapidly at the onset of the test. The presence of various fouling and wetting agents in the mixture accelerated these mechanisms, with effects likely more significant than those given by the sum of the individual agents. Due to the previously assessed wetting potential of SDS, further tests were carried out using the synthetic mixture without SDS. Also, such composition reflects that of a produced water during the central and late period of well life, when surfactants are usually no longer needed for extraction. The results are presented in Fig. 5b. In the absence of surfactants, the MD filtrations achieved a reasonable recovery rates but with a sharp flux decline around 60–70% recovery, due to a combination of organic and inorganic fouling. Moreover, since the very beginning of the filtration, irreversible wetting of the membrane was observed with permeate conductivity increasing throughout the entire test duration.

Therefore, a robust pre-treatment is necessary before the MD step. MD experiments were thus performed with synthetic waters that had previously been oxidized by the Fenton reaction. These tests showed a high level of reproducibility, as presented in Fig. S4 of the SI. Interestingly, significant improvements in the MD performance were accomplished, as shown in Fig. 5c,d. The first Fenton-MD test was carried out with the initial solution containing SDS, in which case wetting was still apparent beginning at roughly 40% recovery. This result marks an improvement compared to the direct application of MD to the untreated synthetic water. For a significant part of the experiment, the water flux was consistent with theoretical expectations and the product quality was high. However, the data also suggest the occurrence of significant concentrations of SDS and organic substances in the solution, even after the Fenton reaction. Actually, as reported in Table 1, the Fenton treatment did not decrease significantly the overall TOC concentration in the solution. This observation may be rationalized considering that the Fenton reaction does not necessarily achieve the complete mineralization of the contaminants, but their transformation to more oxidized organic molecules. In our experiments, however, the Fenton degradation products appeared to be less prone to cause wetting compared to the initial compounds, possibly due to their higher hydrophilicity and lower tendency to interact with the hydrophobic membrane^52–54^. Furthermore, the partial oxidation of SDS tails can significantly decrease its amphiphilic properties decreasing the overall wetting ability of the Fenton treated water^55^. Moreover, the permeate had a significant concentration of TOC (around 90 mgC/L), despite a high solute rejection rate; see also Fig. S3. A possible explanation for this finding is that some volatile components may have escaped oxidation or even that some compounds with high vapor pressure were formed following the degradation of humic acids and other larger molecules, then passing as a distillate through the membrane.

Noteworthy results were obtained when the Fenton-oxidized synthetic water without SDS was filtered in MD. Clearly, the maximum recovery was still governed by scaling, and it reached a value of roughly 65%, related to a final feed TDS concentration of 300 g/L. Remarkably, no significant wetting was observed during these experiments, which may be ascribed to either the higher degradation efficiency of the Fenton reaction on this feed stream or the production of more hydrophilic substances compared to the starting compounds. Interestingly, the Fenton reaction was not inhibited by the high concentration of chloride in the feed solution, suggesting the possibility to apply this pre-treatment step even in complex hypersaline solutions. This finding is also confirmed by the relatively low TOC concentration measured in the oxidized solution (Table 1). Even more importantly, low TOC and TDS concentrations were both associated with the permeate stream. The combined effect given by the partial or complete oxidation of the organics and the absence of SDS in solution strongly improved the efficiency of the desalination process.

Although this preliminary study cannot claim to provide ultimate answers to the problems of produced water treatment by MD, it puts forward important implications for both scientific and applied studies. In particular, our results suggest that: (i) to apply the innovative MD process and achieve high recovery of water from produced water, the nature and the extent of pre-treatment to remove certain organics is crucial; (ii) advanced oxidation, and specifically the Fenton reaction, is a promising pre-treatment option to produce an effluent that is suitable for MD, provided that upstream primary treatments are included to aid this degradation process by removing surfactants and of most of the immiscible compounds; (iii) the goal of the Fenton reaction should be to completely mineralize or at least extensively oxidize the organic contaminants and to minimize the occurrence of volatiles in the effluent. To do so, the process should be optimized in terms of reagent addition and residence time, and properly combined with the downstream polishing step. This downstream separation step, in addition to remove spent iron from the Fenton reaction, should also eliminate all the remaining components that may be hazardous for the MD performance, and the goal could be achieved with a compact multipurpose settling/stripping reactor. Clearly, the individual processes of oxidation and MD, as well as their coupling should be further investigated and optimized, also considering the specific wastewater source and the intended use of the final product.

## Materials and Methods

### Produced water composition

In this study, we used synthetic produced water that contained model contaminants, to obtain the desired value of total organic carbon (TOC) and total dissolved solids (TDS). The composition is based on published values of real produced waters^4,56,57^ and is listed in Table 2 together with the respective TOC and TDS values. Humic acids and a liquid petroleum jelly consisting of paraffins were used as representative compounds for natural dissolved organic matter and oil & grease, respectively^58^. P-xylene, benzene, toluene, and MTBE were selected as representative VOCs^56^, while SDS was used as model surfactant; cyclohexane, and naphthalene were added as representatives of the <C10 and >C10 hydrocarbon fraction, respectively^4,58^; finally, phenol was added because it is generally present in produced waters^2,4^. The TDS in our synthetic water were mostly accounted for by sodium, chloride, and sulfate, because these are the most abundant ions present in typical produced waters^4^. One of the objectives of this study is to investigate the effect of dissolved anions on the effectiveness of the Fenton reaction, mainly sulfate and chlorides, both added at high concentration in the synthetic water. Naturally occurring cations have usually little or no detrimental effects on the Fenton reaction.

### Chemicals and membranes

All the organic contaminants were purchased from Sigma-Aldrich (Italian branch, Milan, Italy). Sodium chloride, sodium sulfate, and sodium bicarbonate were acquired from Carlo Erba (Milan, Italy). Ferrous sulfate (FeSO_4_), hydrogen peroxide solution (30% w/w), HCl and NaOH, were also purchased from Sigma-Aldrich, together with the two polyacrylic acid sodium salts with MW of 45,000 and 5,100, used as antiscalants.

After a set of preliminary MD tests (see Fig. S1 of the Supplementary Information), a commercially available hydrophobic polytetrafluoroethylene (PTFE) membrane (Aquastill, Sittard, Netherlands) was selected and used for all the subsequent experiments. The membrane choice was based on its high water flux, which is a basic requirement to ensure the feasibility of MD. Some of the membrane characteristics, provided by the manufacturer or obtained in the lab, are listed in Table 3. The contact angle was estimated by a sessile drop method using a Drop Shape Analyzer DSA100 (KRÜSS GmbH, Germany), following the deposition of deionized water or petroleum jelly droplets (~15 μL) onto a leveled membrane surface. The value of the angle observed immediately after deposition of the liquid on the membrane is reported. The membrane permeability coefficient was computed by dividing the experimental water flux by the vapor pressure difference across the membrane (*vide infra*).

### Direct contact membrane distillation lab system

Among all the possible MD configurations, direct contact membrane distillation (DCMD) exhibits the simplest and most compact plant characteristics for lab experimentation^59^. In this process, the colder liquid is in direct contact with the membrane at the distillate side, and it is enriched by the water vapor condensation during the process. All the MD tests were performed in DCMD configuration with a lab-scale batch system. The feed and distillate streams were circulated counter-currently on their respective sides of the membrane. A constant cross-flow rate of 1.66 L/min (0.278 m/s cross-flow velocity) was maintained during the tests. The housing cell comprised a 250-mm long, 50-mm wide, and 2-mm deep rectangular channel for a total active membrane area of 125 cm^2^. The flux across the membrane was computed by recording the change in weight of the distillate tank in time through a computer-interfaced balance. Initial volumes of 2.2 L and 1 L were used for the feed and distillate solutions, respectively, unless otherwise stated. On the distillate side, purified water with electrical conductivity below 20 μS/cm was used. The temperature and the conductivity in the distillate tank were measured, respectively, through simple probe thermometers immersed in the tank and by a conductivity meter (COND 7+, XS Instruments, Italy). The temperatures in the feed and distillate tanks were maintained constant throughout the experiment, at respective values of 50 ± 2 and 25 ± 1 °C, by means of a thermostatic water bath and a chiller.

### Filtration experiments: protocol and DMCD model

Different sets of high-recovery membrane distillation tests were performed. (i) The first set included filtration experiments to study the detrimental effect of the organic contaminants under study; in these tests, the feed solution was composed by individual organic compounds at the initial theoretical TOC concentration of 800 mgC/L and by 15 g/L of NaCl. (ii) The second set was performed to investigate the extent of scaling by NaCl and the maximum achievable recovery in the batch tests; in these experiments, the feed solution comprised NaCl only, at concentrations ranging between 15 and 150 g/L, with and without polyacrylate antiscalants, at concentrations (when present) of 30 mg/L (45,000 MW) and 10 mg/L (5,100 MW). (iii) The third set of experiments involved the filtration of the synthetic produced water (see Table 2 for its features), and in some tests the synthetic water did not contain SDS. Furthermore, other tests were performed using as the feed stream the supernatant of the suspensions obtained upon Fenton oxidation, pH adjustment, and settling (v*ide infra*). During these MD experiments, three feed sample were collected in the beginning, in the middle, and at the end of the test. A final sample from the distillate tank was also collected. The TOC was measured with a TOC-V_CSH_ analyzer (Shimadzu, Japan), based on the catalytic combustion method. The analyzer was fed with zero-grade air (Sapio, Italy).

A simplified DCMD model was applied to interpret the experimental data^60^. Briefly, the transmembrane flux, *J*_*w*_ (L m^−2^h^−1^), is proportional to the interfacial water vapor pressure difference, $\Delta {p}_{i}$ (bar), and to the permeability coefficient, *b* (L m^−2^h^−1^bar^−1^), which is in turn a function of the membrane material properties, as well as of the operating temperatures according to the following equation^61^:

$${J}_{w}=b\varDelta {p}_{i}$$

The vapor pressure for pure water, ${p}_{0}$ (Pa), can be estimated using Antoine’s equation:

$${p}_{0}={e}^{23.238-\frac{3841}{(T-45)}}$$

The vapor pressure calculated in this study is based on bulk temperatures of the feed, *T*_*f,b*_ (K), and permeate, *T*_*p,b*_ (K). Bulk temperatures can be used instead of the interfacial ones, by assuming sufficiently low temperature polarization^37^. The feed vapor pressure is also influenced by the coefficient of activity of water, ${a}_{w}$ (−), which depends on the molality *m* (mol/kg) of the feed. This coefficient can be expressed as follows^62^:

$${a}_{w}=1-0.03112\,m-0.001482\,{m}^{2}$$

The permeate molality is considered equal to zero for all the experiments due to the high ion rejection observed. Finally, the vapor tension difference between the feed and the permeate can be expressed as follows:

$$\varDelta {p}_{i}={p}_{f,b}-{p}_{p,b}={a}_{w}{p}_{0,f,b}-{p}_{0,p,b}$$

For each time interval, the value of permeate concentration, *c*_*P*_ (g/L), was computed by a mass balance of the relevant concentrations in the feed, *c*_*F*_ (g/L), and in the distillate tanks. The average rejection of the membranes for each relevant time interval was calculated as:

$$R=1-\frac{{c}_{P}}{{c}_{F}}$$

### Fenton oxidation experiments

The oxidation of the synthetic produced water, with and without SDS, was performed at pH 3 by adding HCl and subsequently 11.62 g FeSO_4_ to achieve a Fe(II) concentration of 19 mM. It is important to add FeSO_4_ in an already acidic medium in order to avoid its premature precipitation as hydroxide. HCl was chosen to adjust the pH because a high concentration of chloride was already present in solution, but the influence of the added acid on the ion balance of the synthetic solution was negligible. To promote organics oxidation, six additions were carried out, each one of a 7.2 mL aliquot of a stock solution of hydrogen peroxide (30%). With one addition every ten minutes, the final H_2_O_2_ concentration was 32 mM and the total duration of an oxidation experiment was one hour. At the end of the reaction, the pH was increased to 8 by addition of NaOH and this step caused the precipitation of Fe(OH)_3_. After allowing for the sedimentation of the precipitate to continue overnight at 4 °C, at least 1.9 L of the supernatant was collected and used as feed solution for the following MD filtration test.

## Supplementary information

supplementary informations
