# Supplementary material for: Desalination of Produced Water by Membrane Distillation: Effect of the Feed Components and of a Pre-treatment by Fenton Oxidation
Source: Sci Rep. 2019 Oct 18;9:14964. doi: 10.1038/s41598-019-51167-z (PMC6802402; doi:10.1038/s41598-019-51167-z)
Supplement: Supplementary file 1 — supplementary informations [file 41598_2019_51167_MOESM1_ESM.pdf]

SUPPLEMENTARY INFORMATION

Desalination of Produced Water by Membrane Distillation: Effect of the Feed Components and of a Pre-treatment by Fenton Oxidation

Francesco Ricceri[1,2], Mattia Giagnorio[1], Giulio Farinelli[1], Giulia Blandini[1], Marco Minella[3], Davide Vione[3], Alberto Tiraferri[1,2]

1: Department of Environment, Land and Infrastructure Engineering, Politecnico di Torino, Corso Duca degli Abruzzi, 24 – 10129 Torino (Italy)

2: CleanWaterCenter@PoliTo, Corso Duca degli Abruzzi, 24 – 10129 Torino (Italy), web: http://cleanwater.polito.it/

3: Department of Chemistry, Università degli Studi di Torino, Via Pietro Giuria 7, 10125 Turin, Italy

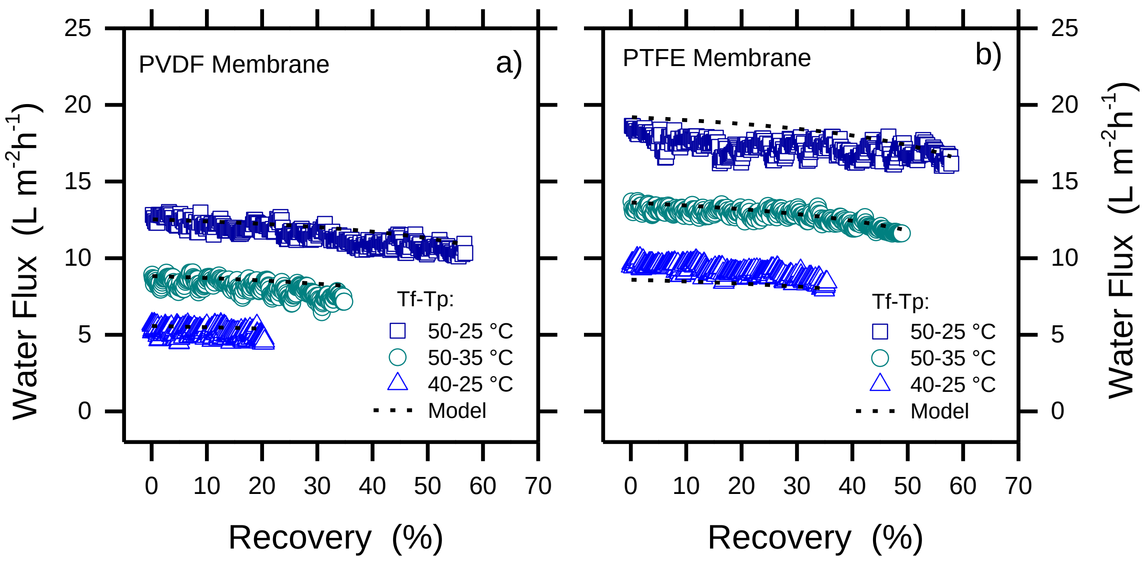

Figure S1. Measured water fluxes in MD filtration tests at medium recovery with a) PVDF and b) PTFE membranes. The concentration of TDS was 100 g/L for all the feed solutions. The tests were conducted at temperatures in the feed/distillate tanks of (squares) 50°C/25°C, (circles) 50°C/35°C, and (triangles) 40°C/25°C. The model for water flux is depicted by black dash lines.

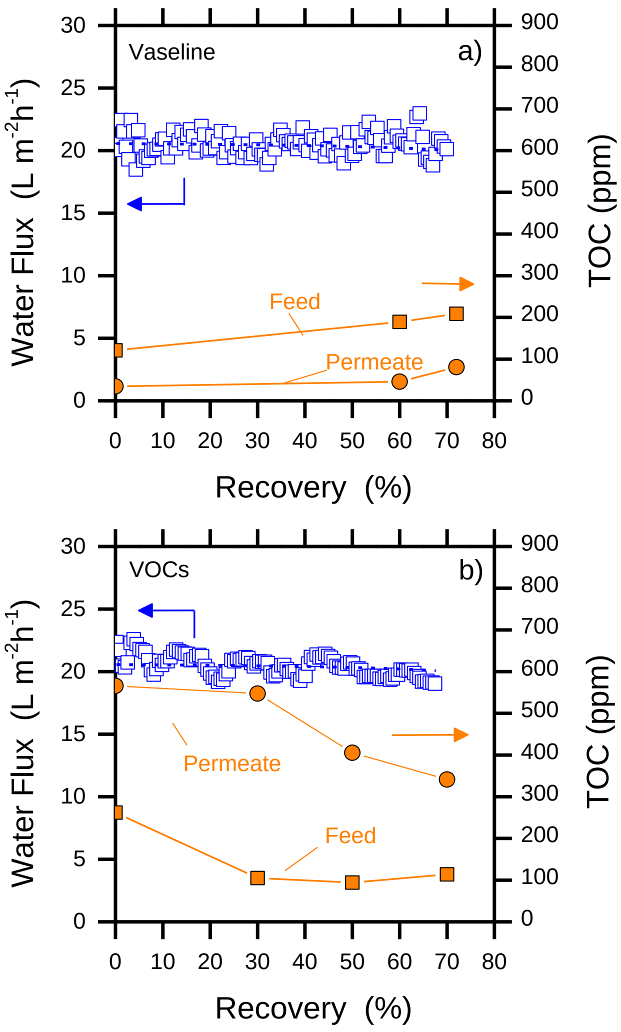

Figure S2. Results of MD filtration tests at high recovery with feed water composed by individual TOC compounds, namely, a) paraffins, b) VOCs. Blue open squares represent the experimental water flux (left Y-axis), while orange solid circles and squares refer to the measured permeate and feed TOC concentrations, respectively (right Y-axis). The model for water flux is depicted by a blue dash line. The initial theoretical total organic carbon was 800 mg_C/L and the concentration of TDS was 15 g/L for all the feed solutions. All the tests were conducted at a feed temperature of 50°C and a distillate temperature of 25 °C, using PTFE membranes.

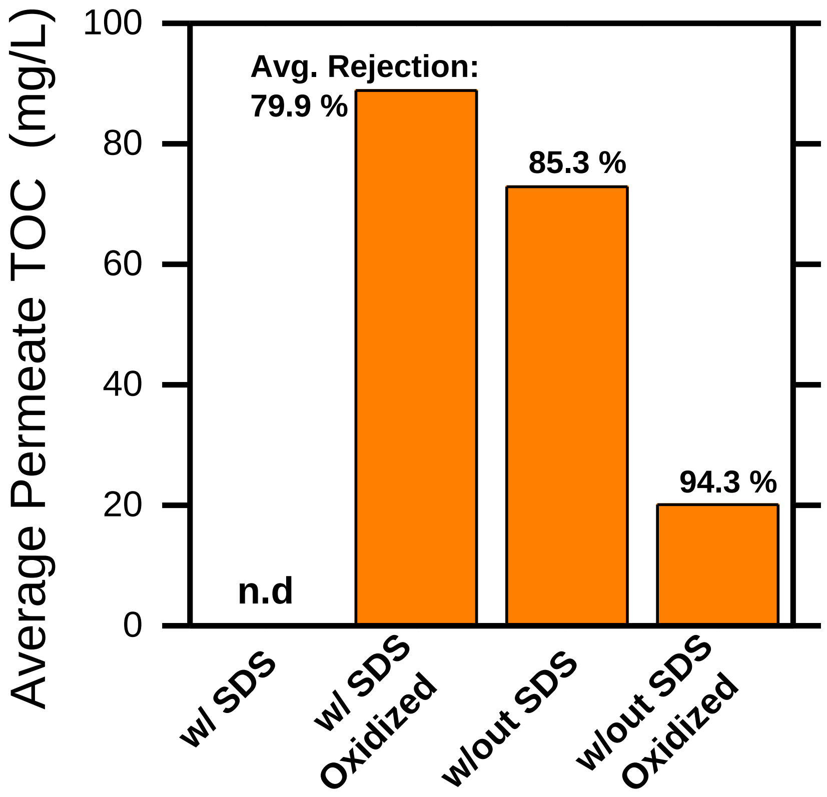

Figure S3. Average TOC concentration in the permeate stream and relative average rejection rates. The values are averages of data recorded during the whole high-recovery test shown in Figure 5 of the main manuscript.

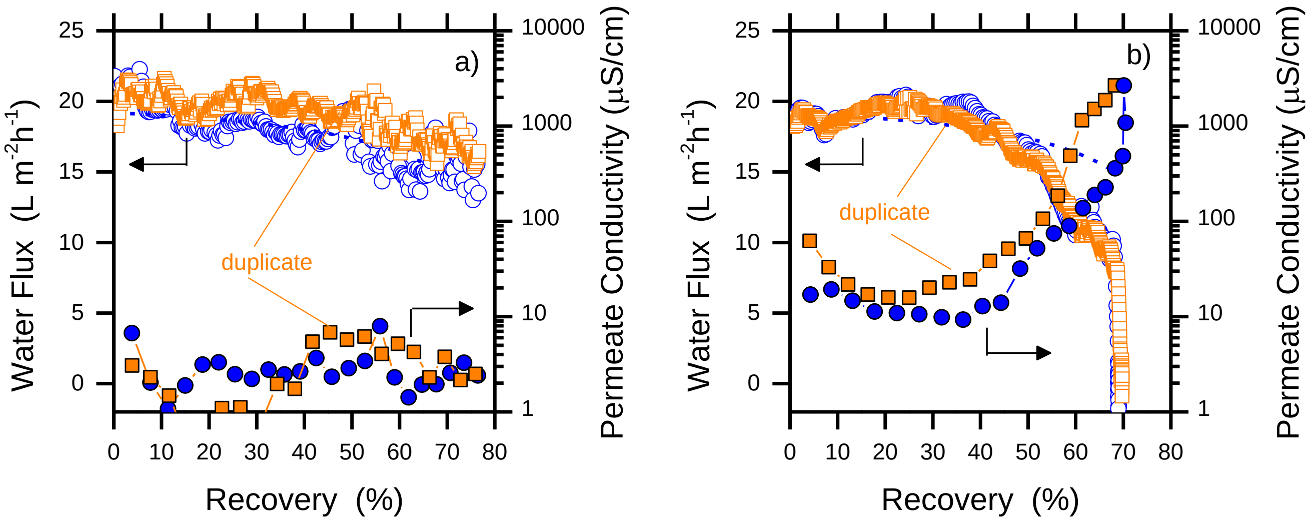

Figure S4. Duplicate experiments for the data presented in the main manuscript in (a) Figure 1c and (b) Figure 5c.

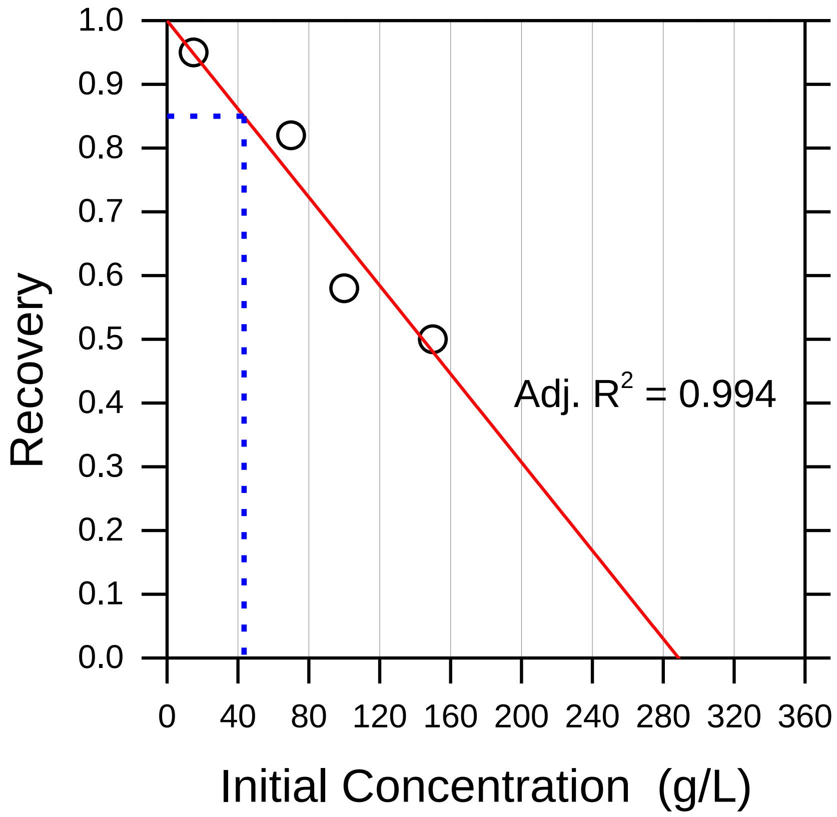

Figure S5. Maximum recovery obtained experimentally as a function of initial NaCl concentration, from the data presented in Figure 3 of the main manuscript. The red line represents the best linear fit with intercept equivalent to 1 (i.e., complete theoretical recovery when no salt is present in the feed solution). At 0% recovery, the concentration is equivalent to $-\frac{1}{slope} = -\frac{1}{0.0035} = 285$ g/L. This value represents the average final NaCl concentration from the various experiments based on the linear fit. For recovery values of 85%, this value is reached with an initial concentration of roughly 43 g/L; see blue dash lines.
